# In Vitro Conservation of Mexican Garlic Varieties by Minimal Growth

**DOI:** 10.3390/plants12233929

**Published:** 2023-11-22

**Authors:** Bibiana Tirado, Víctor Manuel Gómez-Rodríguez, Carlos Iván Cruz-Cárdenas, Lily Xochilt Zelaya-Molina, Humberto Ramírez-Vega, Gabriela Sandoval-Cancino

**Affiliations:** 1 Centro Universitario de los Altos, Universidad de Guadalajara, Av. Rafael Casillas Aceves 1200, Tepatitlán de Morelos 47620, Jal, Mexico; bibiana.tirado9313@alumnos.udg.mx (B.T.); victor.gomez@cualtos.udg.mx (V.M.G.-R.); humberto.rvega@academicos.udg.mx (H.R.-V.); 2 Centro Nacional de Recursos Genéticos-INIFAP, Av. Boulevard de la Biodiversidad 400, Tepatitlán de Morelos 47600, Jal, Mexico; cruz.ivan@inifap.gob.mx (C.I.C.-C.); zelaya.lily@inifap.gob.mx (L.X.Z.-M.)

**Keywords:** garlic, genetic resources, in vitro conservation, osmotic agents

## Abstract

Garlic (*Allium sativum* L.) is one of the 30 crops that are essential for world food; therefore, its conservation should be considered a priority. There are two main plant conservation strategies: in situ and ex situ conservation. Both strategies are important; nevertheless, ex situ field conservation is affected by biotic and abiotic factors. A complementary strategy to preserve garlic germplasm in the medium term is through in vitro culture by minimal growth. The aim of this study was to evaluate the in vitro conservation of three Mexican garlic varieties by minimal growth. Garlic plants obtained from in vitro garlic bulbs were preserved in six culture media at 25, 18, and 5 °C. A randomized design was used and an analysis of the variance of the survival, contamination, and shoot height of the explants was performed at 30, 60, 90, 180, 270, and 365 days of culture. The results showed that the in vitro conservation of Pebeco, Tacátzcuaro Especial, and Huerteño garlic varieties was optimally obtained for one year at 5 °C in a basal Murashige and Skoog (MS) culture medium with 68.46 g L^−1^ sucrose and 36.43 g L^−1^ sorbitol. Thus, the achieved protocol can be adapted to other varieties of garlic for medium-term storage in germplasm banks.

## 1. Introduction

Garlic (*Allium sativum* L.) is a vegetable native to Central Asia and is one of the most important species of the *Allium* genus due to its particular taste and smell, as well as its beneficial properties for human health [1]. Garlic bulbs contain organosulforates, flavanols, saponins, and sapogenins—substances with great potential in medicine and agriculture [2,3]. These compounds have antimicrobial, antioxidant, and flavor-enhancer effects, among others [4].

There are 910 varieties of garlic grown in 35 countries [5], but some of these are only conserved under field conditions and, therefore, are at risk of loss due to biotic and abiotic factors, even during post-harvest [6]. For that reason, it is important to carry out garlic preservation and its genetic resources [7].

Germplasm banks are essential to preserve the genetic diversity of species of agri-food importance, such as garlic. There are 1750 germplasm banks around the world that preserve different species in medium-term and long-term conservation, including elite and local varieties and wild relatives [8]. The preservation of genetic resources is stipulated in international treaties such as the Convention on Biological Diversity and the International Treaty on Plant Genetic Resources for Food and Agriculture [9].

Conservation of sub-orthodox and recalcitrant seeds and vegetatively propagated species, such as garlic, is carried out by in vitro culture techniques [10]. In vitro plant culture enables cells, tissues, and organs to grow in aseptic culture media [11].

Minimal growth is an in vitro culture technique for medium-term plant conservation, from a few months to more than a year, according to the species [12]. Plant growth is slowed by chemical and physical factors. Some of these factors are the decrease of culture medium nutrients, the addition of osmotic agents, growth retardants, mineral oils, low conservation temperature, low light intensity, and shorter photoperiod [13].

This medium-term plant conservation technique has been applied to different species using specific protocols for each one and even for each genotype due to genetic diversity [14]. Regarding garlic, Benke et al. [15] reported 70% survival of the Bhima variety after five months in Murashige and Skoog (MS) culture medium supplemented with sucrose (68.46 g L^−1^) and sorbitol (36.43 g L^−1^). Likewise, Pardo et al. [16] obtained 73% survival of the Boconó clone after seven months of storage in MS medium (25%) containing 45 g L^−1^ of sucrose. Both investigations were performed under standard light and temperature conditions (16 h light photoperiod, 25 μm m^−2^ s^−1^, and 25 ± 1 °C). On the other hand, Hassan et al. [17] preserved Balady and Seds 40 varieties for 15 months at 4 °C in MS medium with sorbitol (72.87 g L^−1^) in dark conditions and obtained survival rates of 35.7% and 90% for each variety, respectively.

Different garlic varieties and their wild relatives are in medium-term and long-term preservation worldwide [18]. However, in most cases, there is a cultivar- or genotype-specific response. In Mexico, there are garlic varieties of agri-food and breeding importance for which there are no in vitro conservation protocols, and they are not stored in a germplasm bank. In this sense, it is vital to generate in vitro conservation protocols because the current conservation of garlic germplasm depends on field regeneration and noncontrolled storage conditions where it is exposed to biotic and abiotic threats, such as unfavorable environmental conditions, pillage, pests, and diseases. Minimal growth provides a medium-term in vitro conservation alternative that allows to safely preserve important germplasm under controlled conditions, efficiently using space and resources. Due to this, the objective of this work was the in vitro preservation of three Mexican garlic varieties by minimal growth.

## 2. Results

### 2.1. Shoot Height

In the case of Pebeco’s explant shoot height, significant differences related to temperature were observed at all periods except at 90 days (*p* < 0.05). In culture media, significant differences were present in this variable at 30, 60, and 365 days (*p* < 0.05). At 365 days, the explant length was shorter in culture medium M1 formulated with Dunstan and Short (BDS) salts and 100 g L^−1^ sucrose (67.54 mm) (Table 1).

On the other hand, significant differences in shoot height of Tacátzcuaro Especial related to conservation temperature were observed at all cultivation periods (*p* < 0.05). Specifically, at 365 days, a shorter length of 89.17 mm was recorded at 25 °C. Also, the effect of culture media was present with reports of significant differences at all cultivation periods (*p* < 0.05). Less growth (78.61 mm) was obtained in culture medium M1 at 365 days (Table 2).

For Huerteño’s, significant differences in shoot height between the tested temperatures were observed at 30, 60, and 90 days with reports of less growth at 5 °C (*p* < 0.05). Also, significant differences in culture media were noted, particularly a shorter length of 49.41 mm in culture medium M1 was recorded at 365 days (Table 3).

**Figure 1:**
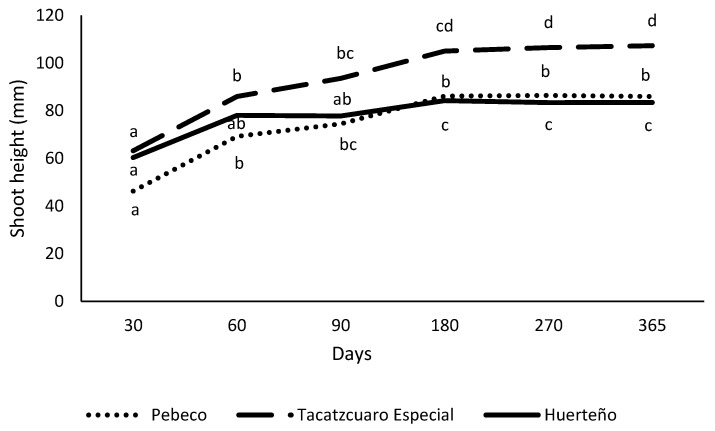
Shoot height development of three garlic varieties during 365 days under minimal growth conditions. The mean values in each evaluation period for each variety that shares the same letter did not differ significantly (*p* < 0.05) according to the Tukey HSD test.

In addition, Figure 1 shows the shoot height development of the three garlic varieties studied during 365 days under minimal growth conditions, where the shoots of Tacátzcuaro Especial showed greater height over time compared with Pebeco and Huerteño.

### 2.2. Survival and Contamination

In the case of Pebeco´s explant survival and contamination, significant differences (*p* < 0.05) linked to temperature and culture medium were observed. At 365 days, higher survival was observed at 5 °C (72.2%) and in culture medium M5 (66.7%). At the same evaluation period, there was less contamination in explants incubated at 5 °C (47.2%) and planted in M4 culture medium (38.9%) (Table 4).

The survival of Tacátzcuaro Especial´s explants showed only significant differences between temperature treatments after 365 days of conservation with reports of a greater rate (95.8%) at 5 °C. In culture media, significant differences were also observed in this variable at all culture periods, except after 365 days (*p* < 0.05). At 365 days, the survival range was 75 to 91% in different culture media (Table 4). Regarding Tacátzcuaro Especial contamination, significant differences linked to storage temperature were observed only at 180 and 270 days (*p* < 0.05). Contamination at 365 days showed no significant differences at 5, 18, and 25 °C (14.6, 27.1, and 29.8%, respectively), although less contamination was present at 5 °C. In culture media, no significant differences were observed for this variable at all periods. At 365 days, the contamination ranged between 16.7 and 33.3% in different culture media, and explants in culture media M3 and M6 showed less contamination (~17%) (Table 4).

**Figure 2:**
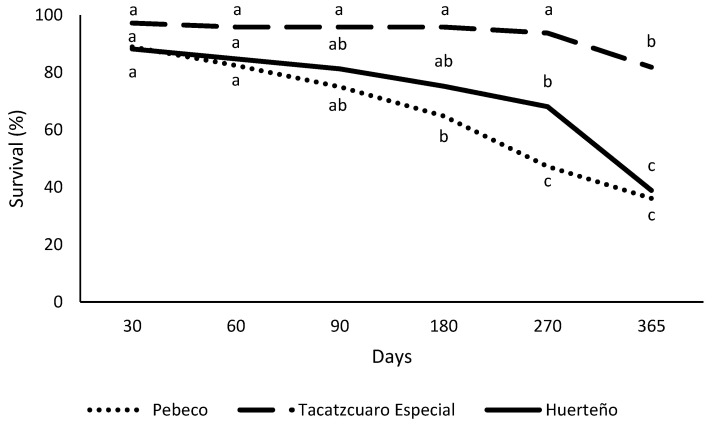
Survival development of three garlic varieties during 365 days under minimal growth conditions. The mean values in each evaluation period for each variety that shares the same letter did not differ significantly (*p* < 0.05) according to the Tukey HSD test.

Significant differences in the survival of Huerteño’s explants were observed at different temperatures after 365 days. Higher survival of 58.3% was reached at 5 °C compared with 18 and 25 °C (33.3 and 25.0%, respectively). In culture media, significant differences were noted only at 180 days, which coincides with a significant survival reduction of Huerteño’s explants (Figure 2). At 365 days, the plant survival range in different culture media was from 16.7 to 54.2% (Table 4).

In the case of shoot contamination, significant differences related to conservation temperature were observed for all cultivation periods of Huerteño plants. At 365 days, less contamination was reported at 5 and 25 °C (43.8%) while higher contamination of 81.3% was reported at 18 °C. In culture media, contamination observed in different culture media was between 50.0 and 66.7% after 365 days of storage under minimal growth conditions (Table 4).

The change in the survival of the three Mexican garlic varieties over time was studied (Figure 2). TacátzcuaroEspecial’s survival was higher at all tested periods compared with the other varieties. Although the obtained results were different between varieties, the survival was constant until culture day 180, when the survival of Pebeco and Huerteño varieties significantly decreased regardless of the temperature and culture medium used.

**Figure 3:**
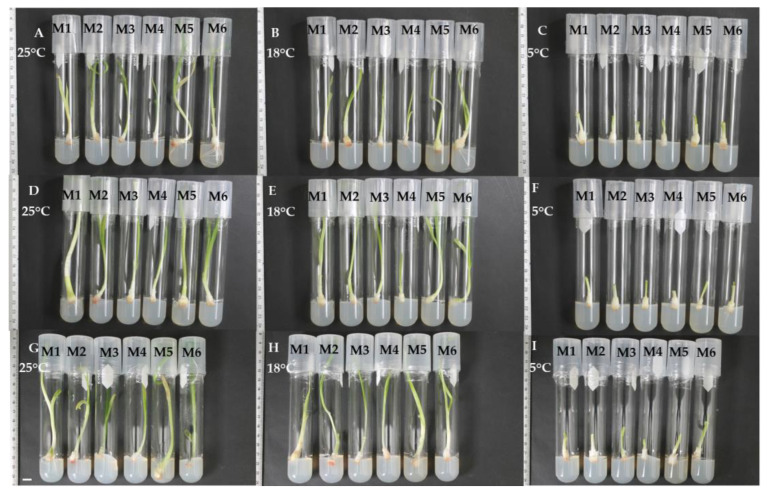
General aspects of minimal growth of garlic in six culture media at 30 days of culture at 25, 18, and 5 °C. (**A**–**C**) Pebeco; (**D**–**F**) Tacátzcuaro Especial; (**G**–**I**) Huerteño. M1: BDS + 100.0 g L^−1^ suc; M2: MS (25%) + 45.0 g L^−1^ suc; M3: MS + 15.0 g L^−1^ mannitol + 15.0 g L^−1^ suc; M4: MS + 72.87 g L^−1^ sorbitol; M5: MS + 68.46 g L^−1^ suc + 36.43 g L^−1^ sorbitol; M6: MS + 30.0 g L^−1^ suc. MS: Murashige and Skoog [19], BDS: Dunstan and Short [20], suc: sucrose. The bar indicates 10 mm.

On the other hand, Figure 3 shows general aspects of the minimal growth morphological effect on three garlic varieties in six culture media at 25, 18, and 5 °C after 30 days of culture. It was notable that plants at 5 °C remained smaller compared with those conserved at the other temperatures.

**Figure 4:**
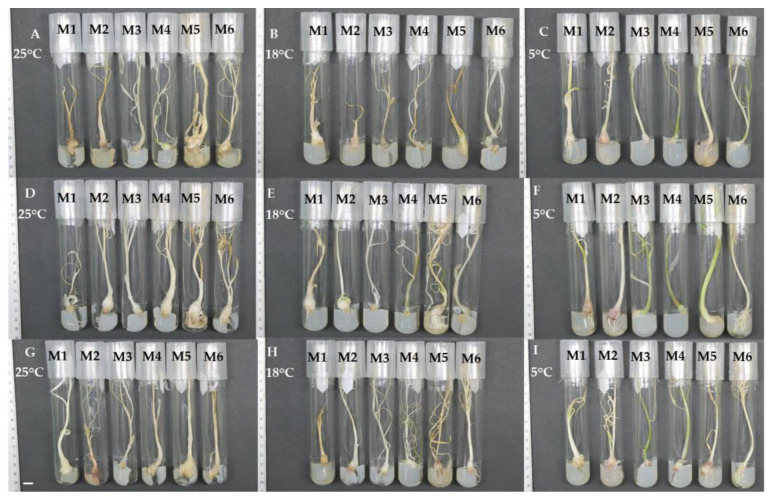
General aspects of minimal growth of garlic in six culture media at 365 days of culture at 25, 18, and 5 °C. (**A**–**C**) Pebeco; (**D**–**F**) Tacátzcuaro Especial; (**G**–**I**) Huerteño. M1: BDS + 100.0 g L^−1^ suc; M2: MS (25%) + 45.0 g L^−1^ suc; M3: MS + 15.0 g L^−1^ mannitol + 15.0 g L^−1^ suc; M4: MS + 72.87 g L^−1^ sorbitol; M5: MS + 68.46 g L^−1^ suc + 36.43 g L^−1^ sorbitol; M6: MS + 30.0 g L^−1^ suc. MS: Murashige and Skoog [19], BDS: Dunstan and Short [20], suc: sucrose. The bar indicates 10 mm.

The in vitro garlic plants of three varieties were kept under minimal growth conditions (six culture media stored at 25, 18, and 5 °C) for 365 days. After this period, better morphological characteristics were observed in plants incubated at 5 °C, such as a bright green color, adequate tissue turgency, and in some cases, bulbification (Figure 4).

### 2.3. Regeneration and Recovery

**Figure 5:**
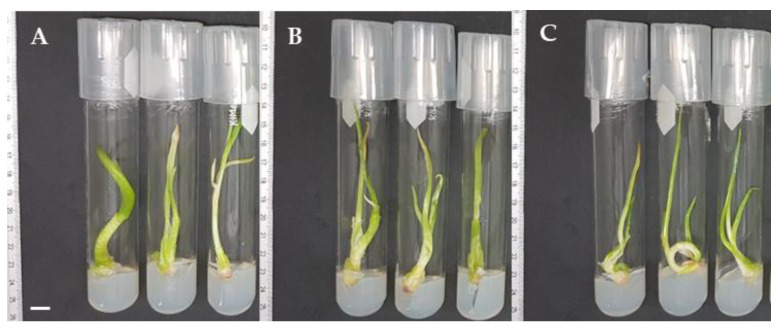
Garlic plants after minimal growth recovery phase (Murashige and Skoog medium with 30 g L^−1^ of sucrose and incubated at 25 °C). (**A**) Pebeco, (**B**) Tacátzcuaro Especial and (**C**) Huerteño. The bar indicates 10 mm.

After 365 days of conservation at 25, 18, and 5 °C in six different culture media, plants stored at 5 °C showed a higher regeneration percentage and recovered their normal growth (Table 5, Figure 5). Specifically, the garlic variety Tacátzcuaro Especial had the highest values of regeneration at 5 °C (Table 5). In the case of all three garlic varieties, regenerated aseptic plants were sufficient as starting material for a future micropropagation step.

## 3. Discussion

Preservation of plant genetic resources is essential to guarantee the well-being of the world’s population with increasing agri-food needs [21]. Also, plant genetic resource conservation contributes to ensuring agricultural and food security for many generations, either, through the use of these resources, to restore specific plant populations or to perform genetic improvement and generate varieties better adapted to the challenges ahead [22].

In this sense, genetic diversity preserved in gene banks must be immediately available [23]. Therefore, minimal growth is an in vitro conservation technique that facilitates the availability, accessibility, and multiplication of plant germplasm [18].

In this study, 5 °C allowed the plant’s conservation time to reach up to 365 days in the Pebeco, Tacátzcuaro Especial, and Huerteño varieties. These results are the first report of a medium-term conservation protocol for these varieties. Cold is one of the main factors that slows plant growth and development [24]. Low temperatures reduce the photosynthetic rate; however, in some cold-sensitive species, they cause depolymerization of chloroplast microtubules [25].

The conservation period of these three varieties in the present study was longer than reported by Benke et al. [15] who preserved the garlic variety Bhima for five months and obtained similar survival with the same culture medium (M5) under standard incubation conditions. Although the M5 culture medium formulated with basal MS medium, sucrose, and sorbitol did not limit the growth of the explants, it favored their survival.

On the other hand, sucrose, sorbitol, and sugars, in general, at high concentrations can cause osmotic stress in plant cells [26]. Osmotic stress affects cell division, morphogenesis, and survival [27]. Gelmesa et al. [28] reported that 0.2 M sorbitol reduced in vitro growth to different degrees in three genotypes of potato (*Solanum tuberosum* L.). In this study, most garlic plants formed bulbs at the three conservation temperatures and mainly at M1 culture medium (100 g L^−1^ sucrose), probably due to a plant survival mechanism strategy induced by osmotic stress. For instance, Alexopoulos and colleagues [29] found that a higher concentration of 8% sucrose in the MS culture medium contributed to enhanced in vitro bulb production in sea lilies (*Pancratium maritimum*). Similarly, in a separate study, the inclusion of 7% sucrose in the MS culture medium was shown to promote bulb formation in elephant garlic (*Allium ampeloprasum* L.) [30]. Moreover, in another investigation conducted by Youssef et al. [31], the use of 9% and 12% sucrose in the MS culture medium resulted in a notable increase in bulb formation for lilies (*Lilium orientalis* cv. “Starfighter”).

Other factors that influence growth and survival during in vitro culture are the explant type, its phenological state, the size and volume of the culture container, and the volume of culture media used [32]. In this sense, during medium-term in vitro conservation, one of the most important variables is shoot height since it is one of the variables that allows choosing the best conditions to extend subculture time [33]. In this study, garlic plants showed their maximum growth in a relatively short period at 25 °C compared with other cultivated species like sweet potato and native species like *Alcantarea nahoumii* (Bromeliaceae) (Leme) J. R. Grant, which were conserved for 4 and even 24 months without significant growth, respectively [34,35]. Nevertheless, shoot growth did stabilize in time, which indicates that all variables and their effects on variety conservation should be considered and evaluated during an appropriate period. Although low temperatures did not limit initial rapid plant growth, they did extend subculture time with higher survival rates in all cases with better morphological characteristics.

Another aspect to take into account when establishing a conservation protocol such as minimal growth is the effect of genotype under specific in vitro conditions. In the present study, different responses can be observed between the three garlic varieties evaluated, whose survival ranges oscillate between 95.8 and 54.2%. This coincides with what was found by Benke et al. [15] who obtained survival percentages between 20 and 70 depending on the garlic genotype evaluated. Therefore, it is necessary to perform the necessary research in order to generate species-specific and sometimes, as in the case of garlic, variety-specific conservation protocols.

One of the main obstacles in in vitro culture is endogenous contamination of the explant [36]. Bacterial growth in the initial stages of in vitro culture is frequent; however, bacterial contamination has also been observed during the multiplication and acclimatization stages [37].

In this study, more bacterial contamination was observed at 18 °C in culture medium M2 (MS medium at 25% supplemented with 45 g L^−1^ sucrose). This could be attributed to the fact that the optimum temperature range for the development of the garlic plant is 12 to 24 °C, and this could also benefit the growth of the natural microbiota of garlic [38].

Regarding culture media, sucrose is the main carbon source in plants in vitro [39]. In case of minimal growth, over time the carbon source is gradually reduced in the culture medium, and the remaining components in it concentrate due to medium dehydration caused by water evaporation. These phenomena cause greater plant stress due to the eventual change in culture medium components availability and osmotic potential. Osmotic stress could be the reason for a greater presence of endogenous garlic microbiota over time. Also in bacteria, sucrose operons are expressed in the presence of sucrose and when other preferred carbon sources are depleted [40].

On the other hand, endophytic microorganisms colonize the vascular tissues of plants and, in most cases, survive surface chemical disinfection [41] and, therefore, can appear months after in vitro establishment, as is the case in the present study. These microorganisms can have a negative or positive effect on plants. In this sense, a bacteriostatic effect has been observed in endophytes isolated from garlic bulbs [42], and endophytic bacteria from garlic roots promote plant growth in vitro [43]. Furthermore, garlic bulbs and garlic oil have different components with antifungal effects [3]. This could be one of the reasons why only major bacterial contamination was observed in two of the three varieties evaluated. Likewise, this could favor the survival of contaminated explants. However, contaminated living plants were discarded for evaluation purposes, and after 365 days of evaluation, they were preserved by minimal growth.

In the present study, plant regeneration after 365 days in conservation by minimal growth was lower than plant survival for all three garlic varieties. Nevertheless, Tacátzcuaro Especial has the highest number of regenerated plants. These results are in accordance with other minimal growth reports in which lower explant regeneration than explant survival at the end of the conservation period was obtained [44,45]. This suggests that plant survival is not sufficient to choose an adequate number of initial plants for medium-term conservation.

The capacity of plant cells to regrow after a medium or long conservation period depends on many factors, like the establishment of adequate regrowth conditions, species, the status of the donor plant or initial material, explant type, and the in vitro conservation technique [22].

## 4. Materials and Methods

### 4.1. Plant Material

Healthy garlic bulbs (*Allium sativum* L.) of Pebeco, Tacátzcuaro Especial, and Huerteño varieties were collected from the field collection in Bajío Experimental Field-INIFAP (National Institute of Agricultural and Livestock Forestry Research), Celaya, Guanajuato, Mexico. These garlic varieties were generated by INIFAP following a traditional breeding program that used the “Taiwan “commercial garlic variety, introduced in 1978 [46].

### 4.2. Initial Explants and Culture Conditions

**Figure 6:**
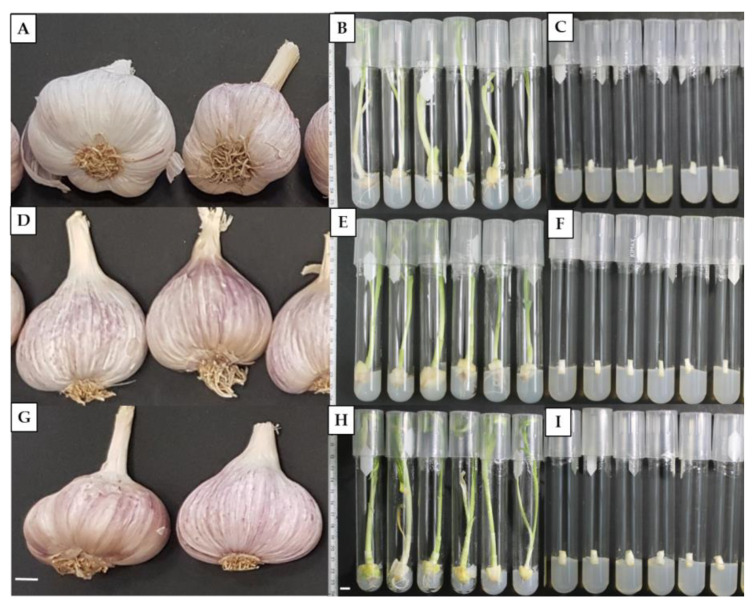
Initial explants and minimal growth experiments. (**A**) Pebeco, (**D**) Tacátzcuaro Especial and (**G**) Huerteño garlic bulbs. (**B**) Pebeco, (**E**) Tacátzcuaro Especial and (**H**) Huerteño plants in MS culture medium supplemented with 30 g L^−1^ sucrose 30 days after in vitro establishment. (**C**) Pebeco, (**F**) Tacátzcuaro Especial and (**I**) Huerteño initial explants in different culture media on day 0 in minimal growth. The bar indicates 10 mm.

Garlic bulbs (Figure 6A,D,G) were divided into individual cloves, which were peeled and washed with commercial detergent and water for 10 min. Garlic cloves were placed in fungicide (1 mL L^−1^, Bravo^®^ 720, Syngenta, Cartagena, Colombia) and fungicide–bactericide (6.25 g L^−1^, Agri-Mycin^®^500, Zoetis, Mexico City, Mexico) solutions for 20 min each with constant stirring. Subsequently, they were disinfected with ethanol at 70% (*v*/*v*) for one minute and with a solution with 1.5% NaOCI for 20 min under a laminar flow cabinet (Labconco, Missouri, USA). After each solution, the cloves were rinsed three times with sterile distilled water and cut into 15 mm long and 10 mm wide pieces. Explants were placed in hormone-free semi-solid Murashige and Skoog (MS) medium (PhytoTech Labs, Kansas, USA) [46] supplemented with 30 g L^−1^ sucrose (PhytoTech Labs, Kansas, USA) and solidified with 9 g L^−1^ agar (PhytoTech Labs, Kansas, USA). The pH medium was adjusted to 5.8 ± 0.1 with sodium hydroxide (NaOH; 1N) before sterilization in an autoclave at 121 °C and 1.3 atm pressure for 20 min. The plant cultures were kept for 30 days in a growth room at a temperature of 25 ± 1 °C, photoperiod of 16 h light/8 h dark, and a photosynthetic photon flux density of 25 μmol m^−2^ s^−1^ provided by white fluorescent light (Figure 6B,E,H).

### 4.3. Minimal Growth Experiment and Data Collection

Aseptic in vitro garlic plants were cut to measure 10 mm in length and 5 mm in diameter and placed on six different culture media with a pH of 5.8 and 9 gL^−1^ of agar (Table 6, Figure 6C,F,I). The initial explants were cultivated for 365 days in growth rooms at a temperature of 5, 18, and 25 ± 1 °C, a photoperiod of 16 h light/8 h dark, and a photosynthetic photon flux density of 25 μmol m^−2^ s^−1^ provided by white fluorescent light.

The experiment was performed using a randomized design, and the experimental unit was an explant in a test tube (25 × 150 mm) containing 10 mL of culture medium. Shoot height, survival, and contamination (visual assessment of bacteria presence) were evaluated in explants at 30, 60, 90, 180, 270, and 365 days. In the case of each temperature treatment, the value is the media obtained from six culture media while for each culture medium, the value is the media obtained from the three tested temperatures. For each treatment, six repetitions were used for Pebeco and eight for Tacátzcuaro Especial and Huerteño varieties, respectively. After 365 days of culture, aseptic surviving plants were removed from the minimum growth conditions and cultured for 30 days for recovery in test tubes containing 10 mL of MS medium supplemented with 0.5 mg L^−1^ of benzyladenine (BA), 30 g L^−1^ of sucrose, and 9 g L^−1^ of agar. The pH medium was adjusted to 5.8 ± 0.1 with NaOH (1N) before sterilization in an autoclave at 121 °C and 1.3 atm pressure for 20 min. Plant cultures were kept for 30 days in a growth room at a temperature of 25 ± 1 °C, photoperiod of 16 h light/8 h dark, and a photosynthetic photon flux density of 25 μmol m^−2^ s^−1^ provided by white fluorescent light.

### 4.4. Statistical Analysis

Statistical analysis of the data was carried out using one-way analysis of variance (ANOVA) and followed by Tukey´s test to reveal significant differences between the means at a level of *p <* 0.05 using the statistical software Statgraphics Centurion, Version 17, Virginia, USA.

## 5. Conclusions

In the present study, optimal conditions for minimal growth conservation of Pebeco, Tacátzcuaro Especial and Huerteño garlic varieties for a one-year period were established. Basal MS culture medium supplemented with sucrose and sorbitol at 5 °C led to the best response for the conservation of three Mexican garlic varieties. With these results, a new alternative was generated for the in vitro preservation of important garlic varieties that did not have a safe and sustainable conservation strategy under controlled conditions.

Despite the number of regenerated plants obtained after a year of minimal growth conservation, based on the survival results, it can be recommended to reduce the storage time from 365 to 180 days in the case of Pebeco and Huerteño and to 270 days for Tacátzcuaro Especial. This strategy will guarantee higher numbers of regenerated plants.

Furthermore, endogenous contamination occurred in Pebeco, Tacátzcuaro Especial, and Huerteño varieties, so it is recommended to carry out additional disinfection treatments during the in vitro process and to isolate and identify garlic endophytic microorganisms to provide valuable information on its potential application. Regardless of contamination rates, regenerated aseptic plants are sufficient as starting material for a future micropropagation step. Results obtained during this study help to choose an adequate number of plants for medium-term conservation in future experiments with different varieties.

Even though this study was carried out with specific Mexican garlic varieties, the resulting conservation alternative can be transferred to other varieties generated from the same commercial variety “Taiwan”; therefore, its application can be considered global.

## Figures and Tables

**Table 1:** Effect of storage temperature and culture media on shoot height (mm) of the Pebeco garlic variety.

	Days					
Temperature	30	60	90	180	270	365
5 °C	21.08 ± 1.04 b	55.04 ± 4.59 b	75.04 ± 6.33 a	106.37 ± 5.95 a	108.28 ± 6.02 a	106.47 ± 6.23 a
18 °C	59.10 ± 4.42 a	81.39 ± 5.83 a	82.69 ± 5.94 a	82.79 ± 6.12 b	81.94 ± 6.20 b	81.97 ± 6.20 b
25 °C	58.50 ± 6.66 a	71.29 ± 7.79 ab	65.70 ± 7.00 a	69.11 ± 7.46 b	68.99 ± 7.41 b	69.49 ± 7.60 b
**Culture Medium**						
M1: BDS + 100.0 g L^−1^ suc	40.37 ± 4.33 abc	57.74 ± 5.41 b	61.60 ± 5.57 a	71.52 ± 8.04 a	69.53 ± 7.86 a	67.54 ± 7.24 b
M2: MS (25%) + 45.0 g L^−1^ suc	48.66 ± 7.16 abc	64.56 ± 6.44 ab	72.45 ± 7.18 a	81.45 ± 8.79 a	76.54 ± 8.10 a	73.96 ± 7.97 ab
M3: MS + 15.0 g L^−1^ mannitol + 15.0 g L^−1^ suc	37.45 ± 5.35 bc	62.91 ± 8.06 ab	70.69 ± 8.83 a	85.44 ± 10.79 a	86.08 ± 10.99 a	86.77 ± 11.14 ab
M4: MS + 72.87 g L^−1^ sorbitol	31.60 ± 5.34 c	60.18 ± 10.76 ab	69.53 ± 11.20 a	88.41 ± 10.74 a	95.60 ± 11.26 a	96.85 ± 11.55 ab
M5: MS + 68.46 g L^−1^ suc + 36.43 g L^−1^ sorbitol	61.80 ± 10.05 a	94.47 ± 9.81 a	91.20 ± 8.70 a	106.41 ± 7.73 a	106.67 ± 7.88 a	105.95 ± 8.08 a
M6: MS + 30.0 g L^−1^ suc	57.47 ± 10.49 ab	75.58 ± 10.72 ab	81.38 ± 11.66 a	83.32 ± 11.84 a	84.00 ± 11.79 a	84.80 ± 11.90 ab

Values of mean ± SE with the same letters per column do not present a significant difference (Tukey *p* < 0.05). MS: Murashige and Skoog [19], BDS: Dunstan and Short [20], suc: sucrose. For each temperature, the value is the media obtained from all six culture media, while for each culture medium, the value is the media obtained from the three tested temperatures.

**Table 2:** Effect of storage temperature and culture media on shoot height (mm) of Tacátzcuaro Especial garlic variety.

	Days					
Temperature	30	60	90	180	270	365
5 °C	22.37 ± 1.03 b	50.30 ± 3.49 c	78.73 ± 5.16 b	109.36 ± 5.05 a	115.20 ± 4.98 a	115.85 ± 5.06 a
18 °C	88.22 ± 4.98 a	116.09 ± 3.65 a	112.76 ± 4.43 a	115.54 ± 3.49 a	116.86 ± 3.57 a	116.57 ± 3.54 a
25 °C	79.95 ± 6.52 a	92.05 ± 6.48 b	89.50 ± 6.35 b	90.36 ± 6.82 b	87.13 ± 6.66b	89.17 ± 6.70 b
**Culture Medium**						
M1: BDS + 100.0 g L^−1^ suc	57.46 ± 8.26 b	69.63 ± 7.85 b	75.32 ± 7.83 c	79.58 ± 7.61 b	77.27 ± 7.65 b	78.61 ± 7.35 b
M2: MS (25%) + 45.0 g L^−1^ suc	74.82 ± 9.29 ab	89.12 ± 7.75 ab	99.16 ± 6.15 abc	112.70 ± 6.68 a	113.25 ± 5.88 a	112.92 ± 5.90 a
M3: MS + 15.0 g L^−1^ mannitol + 15.0 g L^−1^ suc	54.23 ± 7.73 bc	80.19 ± 9.22 ab	87.21 ± 8.52 abc	98.98 ± 8.99 ab	100.18 ± 9.14 ab	101.29 ± 9.27 ab
M4: MS + 72.87 g L^−1^ sorbitol	34.77 ± 4.99 c	73.63 ± 10.13 b	80.66 ± 9.40 bc	98.58 ± 7.85 ab	106.71 ± 8.31 a	107.42 ± 8.42 a
M5: MS + 68.46 g L^−1^ suc + 36.43 g L^−1^ sorbitol	80.96 ± 9.50 a	102.02 ± 6.54 a	106.66 ± 6.07 ab	123.00 ± 3.21 a	124.06 ± 3.14 a	124.66 ± 3.12 a
M6: MS + 30.0 g L^−1^ suc	78.84 ± 10.19 a	102.27 ± 8.44 a	112.97 ± 7.62 a	117.67 ± 7.71 a	116.91 ± 7.89 a	118.29 ± 7.91 a

Values of mean ± SE with the same letters per column do not present a significant difference (Tukey *p* < 0.05). MS: Murashige and Skoog [19], BDS: Dunstan and Short [20], suc: sucrose. For each temperature, the value is the media obtained from all six-culture media, while for each culture medium, the value is the media obtained from the three tested temperatures.

**Table 3:** Effect of storage temperature and culture media on shoot height (mm) of Huerteño garlic variety.

	Days					
Temperature	30	60	90	180	270	365
5 °C	23.26 ± 1.61b	40.13 ± 3.47 b	67.44 ± 6.18 b	89.23 ± 7.62 a	88.26 ± 7.47 a	87.96 ± 7.56 a
18 °C	69.81 ± 7.23 a	101.32 ± 27.29 a	75.64 ± 7.53 ab	73.42 ± 7.65 a	74.66 ± 7.48 a	74.18 ± 7.45 a
25 °C	87.87 ± 6.82 a	92.62 ± 6.68 ab	90.21 ± 6.55 a	89.99 ± 6.73 a	87.19 ± 6.59 a	88.25 ± 6.79 a
**Culture Medium**						
M1: BDS + 100.0 g L^−1^ suc	39.62 ± 7.77 c	44.45 ± 7.75 b	47.85 ± 7.65 b	51.80 ± 8.93 b	49.24 ± 8.31 b	49.41 ± 8.43 b
M2: MS (25%) + 45.0 g L^−1^ suc	61.03 ± 9.01 abc	69.07 ± 8.93 ab	77.04 ± 8.90 ab	86.85 ± 9.89 ab	84.71 ± 9.58 ab	83.20 ± 9.75 ab
M3: MS + 15.0 g L^−1^ mannitol + 15.0 g L^−1^ suc	50.95 ± 9.64 bc	59.20 ± 9.65 ab	64.87 ± 9.72 ab	76.88 ± 10.95 ab	74.85 ± 10.67 ab	75.42 ± 10.91 ab
M4: MS + 72.87 g L^−1^ sorbitol	52.57 ± 9.39 bc	69.38 ± 10.36 ab	80.28 ± 9.61 ab	90.46 ± 10.45 a	89.77 ± 10.21 a	91.25 ± 10.37 a
M5: MS + 68.46 g L^−1^ suc + 36.43 g L^−1^ sorbitol	72.65 ± 11.06 ab	84.42 ± 10.37 ab	97.56 ± 10.30 a	94.04 ± 10.97 a	94.69 ± 10.20 a	95.16 ± 10.40 a
M6: MS + 30.0 g L^−1^ suc	85.06 ± 10.23 a	141.62 ± 52.62 a	98.99 ± 8.53 a	105.26 ± 8.76 a	106.95 ± 8.69 a	106.33 ± 8.63 a

Values of mean ± SE with the same letters per column do not present a significant difference (Tukey *p* < 0.05). MS: Murashige and Skoog [19], BDS: Dunstan and Short [20], suc: sucrose. For each temperature, the value is the media obtained from all six culture media, while for each culture medium, the value is the media obtained from the three tested temperatures.

**Table 4:** Survival and contamination (%) of three garlic varieties at 365 days under minimal growth conditions.

	Pebeco		Tacátzcuaro Especial		Huerteño	
Temperature	Survival	Contam.	Survival	Contam.	Survival	Contam.
5 °C	72.2 ± 7.6 a	47.2 ± 8.4 c	95.8 ± 2.9 a	14.6 ± 5.1 a	58.3 ± 7.2 a	43.8 ± 7.2 b
18 °C	13.9 ± 5.8 b	100.0 ± 0.0 a	81.3 ± 5.7 ab	27.1 ± 6.5 a	33.3 ± 6.9 b	81.3 ± 5.7 a
25 °C	22.2 ± 7.0 b	75.0 ± 7.3 b	68.1 ± 6.9 b	29.8 ± 6.7 a	25.0 ± 6.3 b	43.8 ± 7.2 b
**Culture Medium**						
M1: BDS + 100.0 g L^−1^ suc	22.2 ± 10.1 b	83.3 ± 9.0 a	75.0 ± 9.0 a	33.3 ± 9.9 a	16.7 ± 7.8 a	54.2 ± 10.4 a
M2: MS (25%) + 45.0 g L^−1^ suc	16.7 ± 9.0 b	88.9 ± 7.62 a	88.0 ± 6.9 a	29.1 ± 9.5 a	33.3 ± 9.8 a	66.7 ± 9.8 a
M3: MS + 15.0 g L^−1^ mannitol + 15.0 g L^−1^ suc	22.2 ± 10.1 b	83.3 ± 9.0 a	79.2 ± 8.5 a	16.7 ± 7.8 a	33.3 ± 9.8 a	62.5 ± 10.1 a
M4: MS + 72.87 g L^−1^ sorbitol	44.4 ± 12.1 ab	38.9 ± 11.8 b	75.0 ± 9.0 a	20.8 ± 8.5 a	54.2 ± 10.4 a	50.0 ± 10.4 a
M5: MS + 68.46 g L^−1^ suc + 36.43 g L^−1^ sorbitol	66.7 ± 11.4 a	72.2 ± 10.9 ab	91.3 ± 6.0 a	26.1 ± 9.4 a	54.2 ± 10.4 a	54.2 ± 10.4 a
M6: MS + 30.0 g L^−1^ suc	44.4 ± 12.1 ab	77.8 ± 10.1 a	83.3 ± 7.8 a	16.7 ± 7.8 a	41.7 ± 10.3 a	50.0 ± 10.4 a

Values of mean ± SE with the same letters per column do not present a significant difference (Tukey *p* < 0.05). MS: Murashige and Skoog [19], BDS: Dunstan and Short [20], suc: sucrose, contam.: contamination. For each temperature, the value is the media obtained from all six culture media, while for each culture medium, the value is the media obtained from the three tested temperatures.

**Table 5:** Plant regeneration of different garlic varieties explants after 365 days under minimal growth conditions.

	Pebeco		Tacátzcuaro Especial		Huerteño	
Temperature	Plant Regen. (%)	Number of RegeneratedPlants	Plant Regen. (%)	Number of RegeneratedPlants	Plant Regen. (%)	Number of RegeneratedPlants
5 °C	44.4 ± 8.4 a	16	60.4 ± 7.1 a	29	33.3 ± 6.9 a	16
18 °C	0 ± 0 b	0	14.6 ± 5.1 b	7	2.1 ± 2.1 b	1
25 °C	5.5 ± 3.9 b	2	2.1 ± 2.1 b	1	0 ± 0 b	0
**Culture Medium**						
M1: BDS + 100.0 g L^−1^ suc	16.7 ± 9.0 a	3	20.8 ± 8.4 ab	5	8.3 ± 5.8 a	2
M2: MS (25%) + 45.0 g L^−1^ suc	11.1 ± 7.6 a	2	41.7 ± 10.3 a	10	16.7 ± 7.8 a	4
M3: MS + 15.0 g L^−1^ mannitol + 15.0 g L^−1^ suc	16.7 ± 9.03 a	3	29.1 ± 9.5 ab	7	12.6 ± 6.9 a	3
M4: MS + 72.87 g L^−1^ sorbitol	22.2 ± 10.0 a	4	20.8 ± 8.5 ab	5	12.6 ± 6.9 a	3
M5: MS + 68.46 g L^−1^ suc + 36.43 g L^−1^ sorbitol	16.7 ± 9.0 a	3	39.1 ± 10.4 ab	9	12.6 ± 6.9 a	3
M6: MS + 30.0 g L^−1^ suc	16.7 ± 9.0 a	3	4.1 ± 4.1 b	1	8.3 ± 0.8 a	2

Values of mean ± SE with the same letters per column do not present a significant difference (Tukey *p* < 0.05). MS: Murashige and Skoog [19], BDS: Dunstan and Short [20], suc: sucrose, regen.: regeneration. For each temperature, the value is the media obtained from all six culture media, while for each culture medium, the value is the media obtained from the three tested temperatures.

**Table 6:** Culture media composition used for garlic minimal growth.

Culture Media	Mannitol (g L^−1^)	Sucrose (g L^−1^)	Sorbitol (g L^−1^)
M1: BDS	0.0	100.0	0.0
M2: 25% MS	0.0	45.0	0.0
M3: 100% MS	15.0	15.0	0.0
M4: 100% MS	0.0	0.0	72.87
M5: 100% MS	0.0	68.46	36.43
M6: 100% MS (control)	0.0	30.0	0.0

MS: Murashige and Skoog [19]; BDS: Dunstan and Short [20].

## Data Availability

Data are contained within the article.
